# Draft Genome Assembly and Differential Expression of *MpPPO1* and *MpPPO2* Across Green-Fruit Development in Ecuadorian *Musa paradisiaca* L.

**DOI:** 10.3390/plants15152329

**Published:** 2026-07-29

**Authors:** Víctor Huebla-Concha, Nicolás Cruz-Rosero, Jaime Morante-Carriel, Mercedes Carranza-Patiño, Roque Bru-Martínez, Laura Morante

**Affiliations:** 1 Biology Laboratory, Higher Polytechnic School of Chimborazo (ESPOCH), Panamericana Sur, km. 1 ½, Riobamba 060155, Chimborazo, Ecuador; 2 Department of Experimental Biology, University of Jaén, Campus Las Lagunillas s/n, 23071 Jaén, Spain; 3 Molecular Biology Laboratory, Quevedo State Technical University (UTEQ), Central Campus, Av. Quito km. 11/2 vía Santo Domingo de los Tsáchilas, Quevedo 120550, Los Ríos, Ecuadormcarranza@uteq.edu.ec (M.C.-P.); 4 Plant Proteomics and Functional Genomics Group, Department of Biochemistry and Molecular Biology and Soil and Agricultural Chemistry, Faculty of Science, University of Alicante, Carretera San Vicente del Raspeig s/n, 03690 San Vicente del Raspeig, Alicante, Spain; roque.bru@ua.es; 5 Escuela de Nutrición y Dietética, Facultad de Ciencias de la Rehabilitación y Calidad de Vida, Universidad San Sebastián, Campus Patagonia, Lago Panguipulli, Puerto Montt 1390, Región de Los Lagos, Chile; laura.morante@uss.cl

**Keywords:** banana postharvest, enzymatic browning, gene expression, *Musa paradisiaca*, *MpPPO1*, *MpPPO2*, Oxford Nanopore sequencing, polyphenol oxidase

## Abstract

Ecuador ranks among the world’s leading banana exporters; however, postharvest enzymatic browning, driven by polyphenol oxidases (PPOs), remains an important determinant of fruit quality loss and reduced market value. The genes encoding PPOs in *Musa paradisiaca* L., a triploid hybrid (AAB) widely cultivated in Ecuador, have not previously been described at the genomic or transcriptional level. The objective of this study was to generate a preliminary de novo draft assembly of Ecuadorian *M. paradisiaca*, identify PPO-coding loci recovered in this assembly, and compare their relative expression across three field-defined green-fruit developmental stages: early, intermediate, and physiologically mature-green. High-molecular-weight genomic DNA sequencing was performed using the Oxford Nanopore MinION Mk1B platform, and 13,239 contigs were obtained by de novo assembly with Flye v2.9.6. Two intronless PPO-coding loci, designated *MpPPO1* and *MpPPO2*, were identified and deposited in GenBank under accessions PX565008 and PX565009. qRT-PCR showed significantly higher *MpPPO1* expression at the physiologically mature-green stage, whereas *MpPPO2* showed only a non-significant trend toward higher expression at the intermediate stage. These profiles indicate transcriptional divergence and identify *MpPPO1* as a candidate associated with late green-fruit development or physiological maturity. However, because stage classification relied on a qualitative field harvest index and neither controlled postharvest ripening nor quantitative maturity, PPO activity, or browning measurements were performed, the results do not demonstrate ripening-induced activation, functional subfunctionalization, or causality in browning. These findings provide a preliminary molecular basis for future functional and postharvest studies in commercial banana cultivars.

## 1. Introduction

Banana (*Musa* spp.) is one of the most economically important tropical crops worldwide and is a strategic export commodity for Ecuador, a country that consistently ranks among the leading global suppliers. Despite this commercial relevance, postharvest losses remain a persistent challenge, and enzymatic browning of the fruit peel and pulp is among the principal drivers of quality degradation during harvesting, transport, storage, and industrial processing [1,2]. This phenomenon is predominantly mediated by polyphenol oxidases (PPOs), copper-containing enzymes that catalyze the oxidation of phenolic substrates into reactive quinones, which subsequently polymerize into dark pigments.

From a functional perspective, PPOs are classified into three catalytic types—tyrosinase, laccase, and catechol oxidase—with plants being the primary source of catechol oxidase activity [3]. Beyond their role in browning, PPOs participate in secondary metabolism, plant defence against herbivores and pathogens, and stress responses; their notable presence in some lineages and apparent absence in others—such as *Arabidopsis thaliana* (L.) and certain green algae—supports an ecological rather than a strictly primary-metabolic function [4]. Recent genomic and multi-omics studies have further highlighted the importance of enzymatic browning and polyphenol oxidase (PPO) gene family diversification in postharvest quality and crop improvement strategies [5]. In addition, the number of PPO gene-family members varies considerably across species and is not correlated with genome size, suggesting lineage-specific expansions driven by adaptation.

In recent years, PPO gene families have been systematically characterized in various commercially important species, including *Populus trichocarpa* Torr. & A.Gray ex Hook. [3], *Olea europaea* L. [6], *Paulownia fortune* (Seem.) Hemsl. [7], artichoke [8], lettuce [9] and *Musa acuminata* Colla, where ten MaPPO genes were identified, and it was determined that MaPPO1 and MaPPO6 constitute the main candidates associated with fruit browning [10]. A chromosomal-scale genome assembly of *Musa beccarii* N.W.Simmonds revealed extensive rearrangements within the *Musaceae* lineage [11].

The present analysis focused on *MpPPO1* and *MpPPO2* because these were the two full-length PPO-coding loci recovered without internal gaps in the low-coverage draft assembly and because their closest homologues in *M. acuminata* Colla had been linked to fruit browning. Therefore, the study was intentionally restricted to the recovered gene-level loci rather than to a complete genome-wide PPO-family inventory.

Most of the available genomic resources for bananas have been generated for diploid genotypes of *M. acuminata* Colla (genome A) or *Musa balbisiana* Colla (genome B) [12,13], which are the ancestors of most cultivated varieties. *Musa paradisiaca* L., a triploid AAB hybrid resulting from *M. acuminata* Colla × *M. balbisiana* Colla, exhibits marked resistance to diseases and pests [14]. To date, no previous study has sequenced and assembled the *M. paradisiaca* genome using Ecuadorian germplasm. The lack of knowledge regarding the expression of its polyphenol oxidase (PPO)-encoding genes limits the application of molecular strategies to reduce browning in this economically important cultivar.

Long-read sequencing technologies such as the Oxford Nanopore MinION system effectively allow the sequencing of complex plant genomes that are more complicated than those sequenced with short-read platforms [15,16]. Combined with high-resolution assemblers such as Flye [17], these tools enable de novo characterization of non-model plant genomes with relatively modest computational resources.

In this context, the present study was designed with three specific objectives: (i) to generate the first de novo genome assembly of *Musa paradisiaca* L. from Ecuador using Oxford Nanopore long-read sequencing and the Flye assembler; (ii) to identify, annotate, and phylogenetically contextualize the PPO-coding genes recovered in this assembly; and (iii) to quantify the relative expression of the identified MpPPO genes across three field-defined green-fruit developmental stages (early, intermediate, and physiologically mature-green) by qRT-PCR. *MpPPO1* and *MpPPO2* were selected as candidate genes for future functional studies of PPO regulation and enzymatic browning. The expression component should be interpreted as an exploratory comparison among green-fruit developmental categories and not as a controlled postharvest ripening experiment.

## 2. Results

### 2.1. Sequencing Output and Read-Quality Metrics

Nanopore sequencing of *M. paradisiaca* genomic DNA on the MinION Mk1B platform (FLO-MIN106 R9.4.1 flow cell, SQK-RAD004 kit) generated a total of 1.27 × 10^6^ reads with an estimated yield of 4.79 Gb of raw signal. Following Guppy v6.4.6 HAC base-calling, ~3.7 Gb of nucleotide sequence was obtained and exported as 210 FASTQ files containing 836,455 reads after quality filtering (Q ≥ 8, length ≥ 1000 bp), corresponding to 2.83 × 10^9^ bases. The mean per-read quality score was Q8.9, with a median of Q8.8, values that fall within the expected range for standard R9.4.1 chemistries [18]. Reads below the quality threshold (Q < 8.0) were discarded before assembly.

### 2.2. Genome Assembly of Musa paradisiaca

De novo assembly of the filtered reads was performed using Flye (v2.9.6+galaxy0). Assembly and quality control metrics, as well as the results of the comparison with the *M. acuminata* Colla DH-Pahang reference genome using QUAST, are presented in Table 1. The complete assembly was deposited in DDBJ/ENA/GenBank under accession number JBUBWB000000000.1, in accordance with NCBI taxonomic criteria for hybrid cultivars derived from *M. acuminata* Colla × *M. balbisiana* Colla, and is formally designated as the *Musa* Gros Michel hybrid cultivar.

### 2.3. Identification and Annotation of PPO-Coding Genes

Local BLASTn (https://blast.ncbi.nlm.nih.gov/Blast.cgi accessed on 21 July 2024) alignment of the *M. paradisiaca* L. assembly against previously reported *M. acuminata* Colla PPO sequences [10] identified two PPO-coding loci, designated MpPPO1 and MpPPO2. Their structural annotation and main characteristics are presented in Table 2. The genomic, CDS, and predicted protein sequences were deposited in GenBank under accession numbers PX565008 (MpPPO1) and PX565009 (MpPPO2).

### 2.4. Phylogenetic Relationships of MpPPO Genes

**Figure 1:**
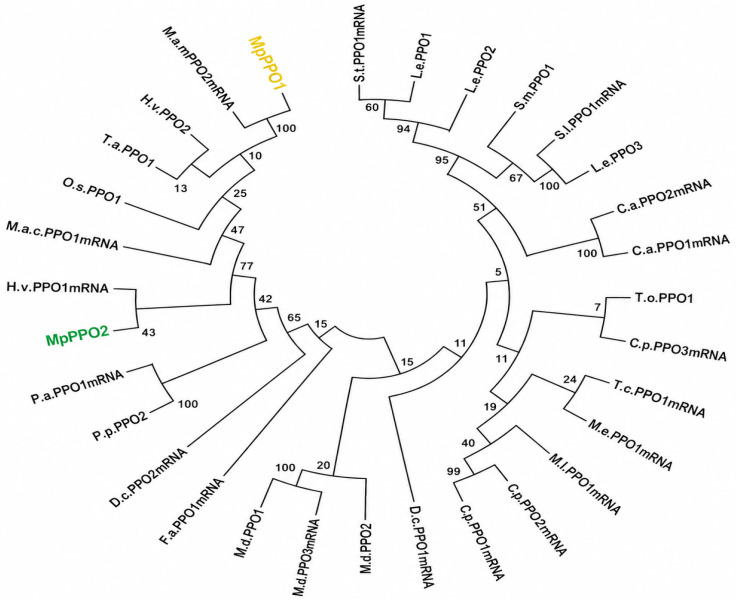
Neighbor-Joining phylogenetic tree of 31 PPO nucleotide sequences from 20 plant species (500 bootstrap replicates). Species abbreviations: T.a: *Triticum aestivum* (wheat); T.c: *Theobroma cacao* (cocoa); T.o: *Taraxacum officinale* (dandelion); S.t: *Solanum tuberosum* (potato); S.m: *Solanum melongena* (eggplant); S.l: *Solanum lycopersicum* (tomato); P.p: *Prunus persica* (peach); P.a: *Prunus armeniaca* (apricot); O.s: *Oriza sativa* (rice); M.a.m: *Musa acuminata* subsp. *malaccensis* (banana); M.a.c: *Musa acuminata* Cavendish (banana); M.e: *Manihot esculenta* (cassava); M.i: *Mangifera indica* (mango); M.d: *Malus domestica* (apple tree); L.e: *Lycopersicon esculentum* (tomatillo); H.v: *Hordeum vulgare* (barley); F.a: *Fragaria ananassa* (strawberry); D.c: *Daucus carota* (carrot); C.a: *Coffea arabica* (coffee); C.p: *Carica papaya* (papaya). The genes highlighted in green (*MpPPO2*) and orange (*MpPPO1*) correspond to their expected positions in the phylogenetic tree.

A Neighbor-Joining phylogenetic tree was reconstructed from 31 PPO nucleotide sequences representing agronomically relevant species (wheat, barley, rice, banana, apple, strawberry, tomato, eggplant, potato, and others), with 500 bootstrap replicates (Figure 1). The tree resolved 19 subgroups, and both *MpPPO1* and *MpPPO2* clustered within the clade containing *M. acuminata* Colla subsp. malaccensis. *MpPPO1* showed the closest evolutionary proximity to *M. a. malaccensis* PPO, whereas *MpPPO2* was positioned at a greater phylogenetic distance within the same clade, consistent with paralogue divergence. This pattern should be interpreted as phylogenetic and expression-level divergence rather than as demonstrated functional subfunctionalization.

### 2.5. Relative Expression of MpPPO1 Across Green-Fruit Developmental Stages

**Figure 2:**
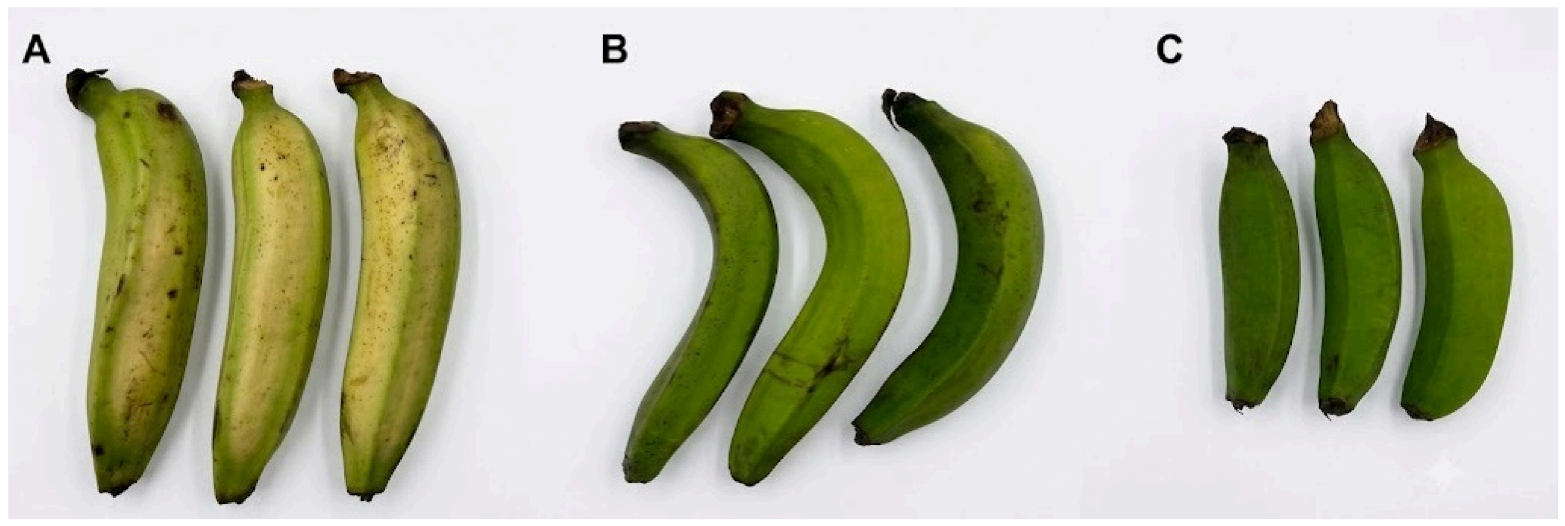
Representative fruits of *M. paradisiaca* L. corresponding to the three field-defined green-fruit developmental stages used in this study. (**A**) Advanced developmental stage, field-classified as physiologically mature-green; (**B**) intermediate green-fruit developmental stage; (**C**) early green-fruit developmental stage. The images document external appearance only and do not constitute quantitative measurements of physiological maturity or consumption ripeness.

**Figure 3:**
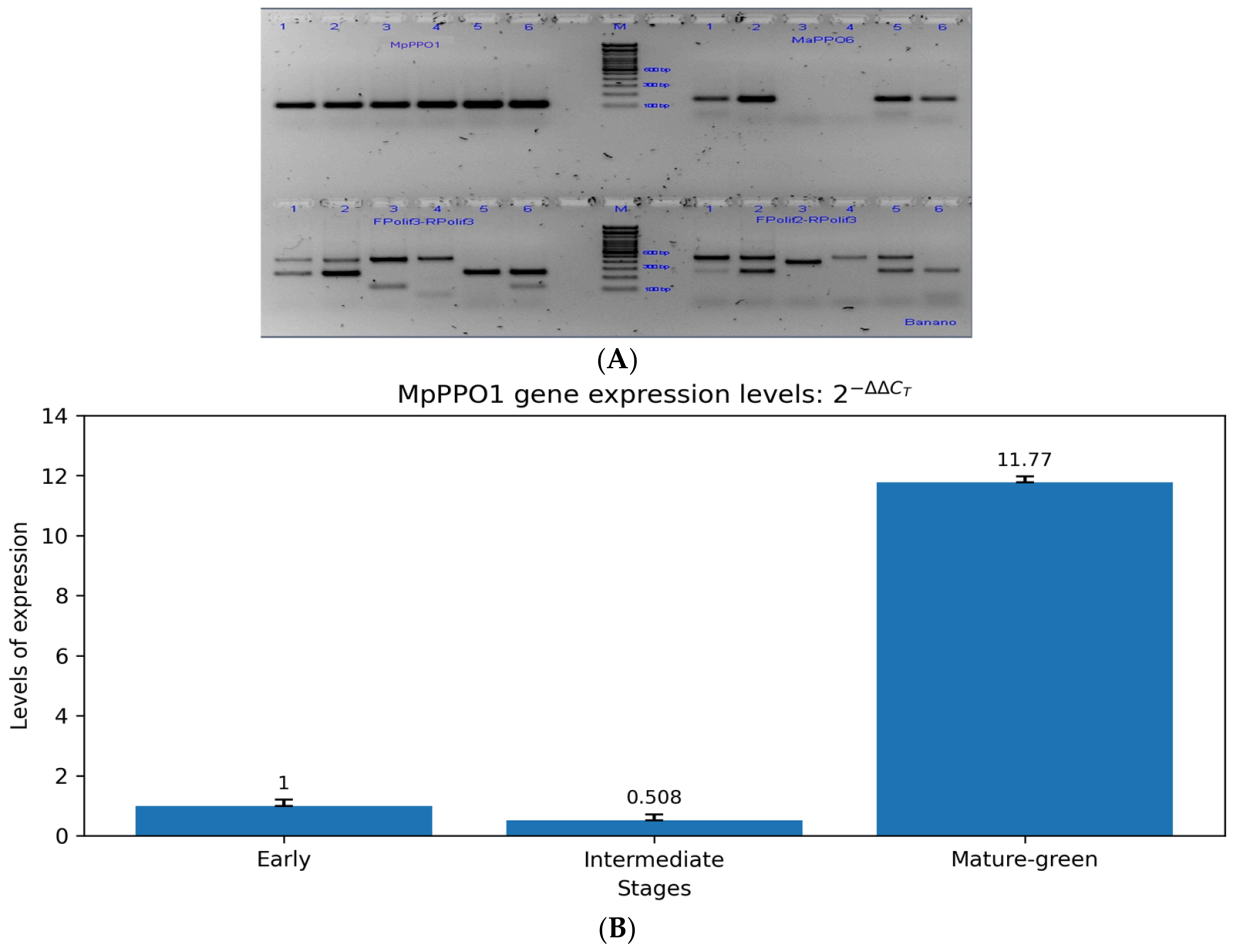
(**A**) Agarose gel electrophoresis (1%) of endpoint-PCR products corresponding to *MpPPO1* (expected amplicon: 112 bp). MM, molecular-weight marker; CN, negative control. (**B**) Relative expression of *MpPPO1* across the early, intermediate, and physiologically mature-green developmental stages of *M. paradisiaca* fruit. Values are expressed as 2^−ΔΔCT^ (mean of three biological replicates per stage) relative to the early-stage calibrator.

The three field-defined green-fruit developmental stages depicted in Figure 2—early, intermediate, and physiologically mature-green—were used for the gene-expression analyses described below. Endpoint PCR amplification of *MpPPO1* cDNA yielded a single, well-defined band of the expected size (112 bp) on 1% agarose gels in all three stages (three biological replicates per stage), confirming transcriptional activity throughout green-fruit development (Figure 3A). Quantitative RT-PCR analysis, using *RPS2* as the endogenous reference gene and the early stage as the calibrator, revealed developmental-stage-associated expression differences (Figure 3B; Table S1). The highest relative expression (2^−ΔΔCT^) of *MpPPO1* was observed at the physiologically mature-green stage (mean = 11.770), followed by the early stage (mean = 1.000), whereas the intermediate stage exhibited the lowest transcript level (mean = 0.508). One-way analysis of variance on ΔCT values revealed a statistically significant effect of developmental stage on *MpPPO1* expression (F2,6 = 14.22, *p* = 0.0053). Tukey’s HSD test indicated that the physiologically mature-green stage differed significantly from both the early (*p* = 0.022) and intermediate (*p* = 0.005) stages, whereas no statistically significant difference was found between the early and intermediate stages (*p* = 0.391). These results demonstrate higher *MpPPO1* transcript abundance in the field-classified physiologically mature-green category. Because postharvest ripening and browning were not experimentally monitored, this pattern is interpreted as an association with late green-fruit development or physiological maturity rather than as evidence of ripening-induced activation or a causal role in browning.

The complete cycle-threshold (Ct), ΔCt, ΔΔCt and 2^−ΔΔCt^ dataset, by biological replicate, underlying the MpPPO1 relative-expression results shown in Figure 3B is provided as Table S1 in the Supplementary Materials in order to avoid duplication of the same summary data in both a table and a figure in the main text.

### 2.6. Relative Expression of MpPPO2 Across Green-Fruit Developmental Stages

**Figure 4:**
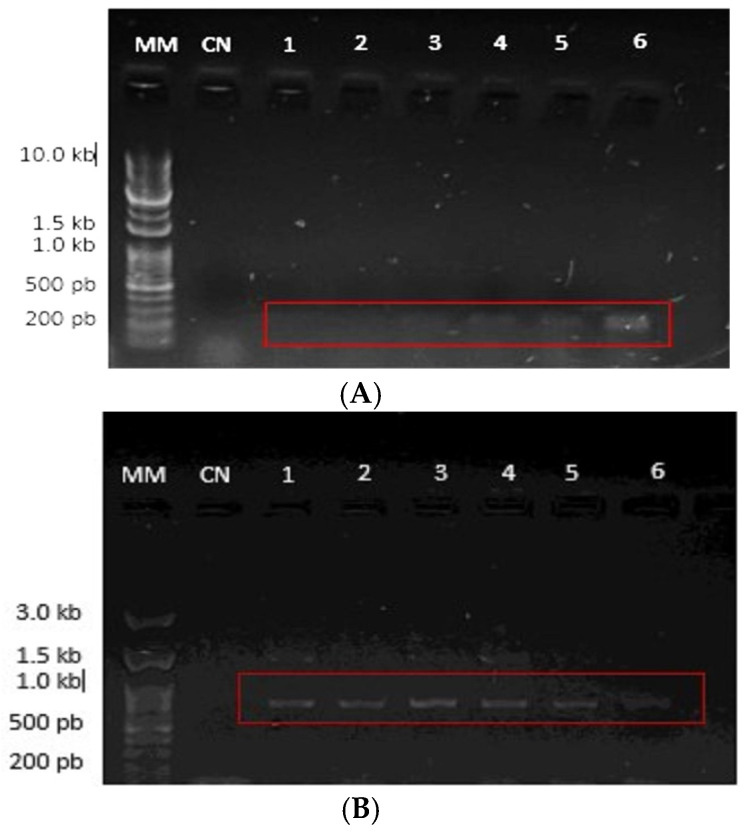
(**A**) Agarose gel electrophoresis (1%) of endpoint-PCR products corresponding to *MpPPO2* (expected amplicon: 237 bp, lanes 1–6, red rectangle). MM, molecular-weight marker; CN, negative control. (**B**) Agarose gel electrophoresis (1%) of endpoint-PCR products corresponding to the ndhB chloroplast fragment (expected amplicon: ~721 bp, lanes 1–6, red rectangle), used as an internal quality control of cDNA synthesis. MM, molecular-weight marker; CN, negative control.

**Figure 5:**
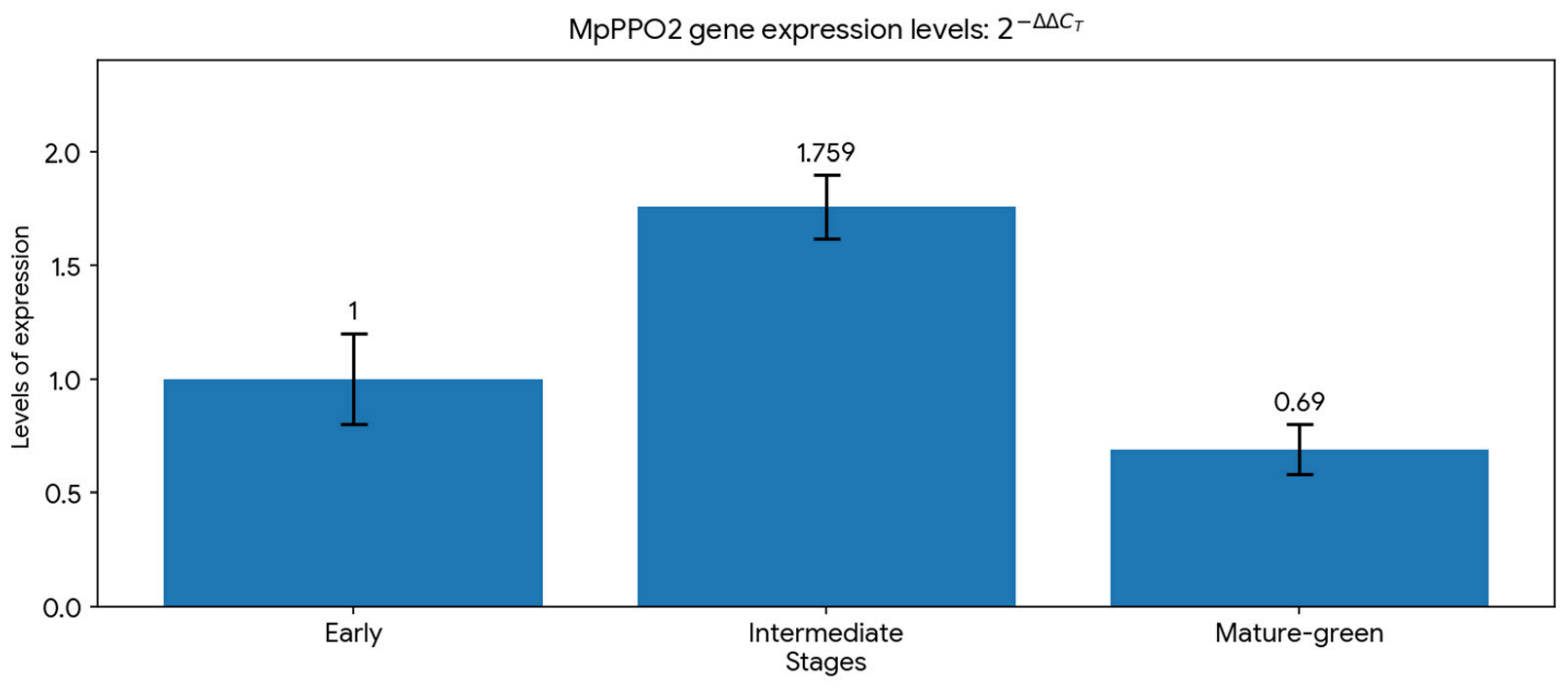
Relative expression of *MpPPO2* across the early, intermediate, and physiologically mature-green developmental stages of *M. paradisiaca* fruit. Values are expressed as 2^−ΔΔCT^ (mean of three biological replicates per stage) relative to the early-stage calibrator.

Endpoint PCR amplification of *MpPPO2* cDNA yielded clear bands of the expected size (237 bp) in the early and intermediate green-fruit stages, whereas amplification was markedly weaker in the physiologically mature-green stage, consistent with lower transcript abundance (Figure 4A,B). Quantitative RT-PCR confirmed this trend (Figure 5; Table S2): *MpPPO2* displayed its highest relative expression at the intermediate stage (mean = 1.759), followed by the early stage (mean = 1.000), while the physiologically mature-green stage exhibited the lowest transcript level (mean = 0.690). All Ct values for both *MpPPO1* and *MpPPO2* were below 40, confirming amplification in all replicates.

In contrast to *MpPPO1*, one-way ANOVA on ΔCt values did not detect a statistically significant effect of developmental stage on *MpPPO2* expression (F2,6 = 1.88, *p* = 0.232), and no pairwise comparison reached significance in the Tukey HSD test (*p* > 0.05 in all cases). The observed trend—with a maximum at the intermediate stage and lower expression at the physiologically mature-green stage—should therefore be interpreted as descriptive rather than conclusive and requires confirmation with a larger number of biological replicates (Section 3.6).

The complete cycle-threshold (Ct), ΔCt, ΔΔCt and 2^−ΔΔCt^ dataset, by biological replicate, underlying the MpPPO2 relative-expression results shown in Figure 5 is provided as Table S2 in the Supplementary Materials, in order to avoid duplication of the same summary data in both a table and a figure in the main text.

Taken together, the two PPO-coding genes identified in *M. paradisiaca* exhibited contrasting expression profiles across the field-defined green-fruit developmental stages. *MpPPO1* showed markedly higher expression at the physiologically mature-green stage, whereas *MpPPO2* showed a non-significant maximum at the intermediate stage. This contrast is compatible with different transcriptional regulation of the two paralogues, but it does not demonstrate functional subfunctionalization, ripening-specific regulation, or a causal contribution to browning.

## 3. Discussion

### 3.1. Genome Assembly of M. paradisiaca L. in a Comparative Context

This study presents the first de novo genome assembly of *M. paradisiaca* L. derived from Ecuadorian germplasm. Using Oxford Nanopore long-read sequencing on a FLO-MIN106 R9.4.1 flow cell with the SQK-RAD004 rapid library and the Flye assembler [17], 13,239 contigs were reconstructed, covering 42.86% of the *M. acuminata* Colla DH-Pahang reference genome. Although the contiguity obtained (N50 = 44,493 bp) is lower than the chromosome-scale assemblies recently reported for other *Musa* species, it is consistent with early-draft assemblies generated from moderate sequencing depth (estimated at ~5×). In comparison, the assembly of the *M. beccarii* N.W.Simmonds genome using Nanopore sequencing data and the assemblers Flye, Canu, Hifiasm, and NextDenovo produced total assembly sizes ranging from 607 Mb (NextDenovo) to 816 Mb (Canu), with a BUSCO completeness score of 98.4% [11]. The lower genome fraction observed in our study most likely reflects the limited sequencing depth achieved on a single MinION flow cell and the inherent heterozygosity of the triploid AAB genome of *M. paradisiaca* L. rather than an intrinsic limitation of the assembler. Accordingly, the assembly reported here should be regarded as a reference draft, valuable for gene identification and comparative analyses within the Musaceae, but not yet as a chromosome-scale resource.

Regarding read-quality metrics, average Nanopore read qualities of approximately Q11 have been reported for plant genome assemblies [18], whereas our dataset yielded a mean quality score of Q8.9, consistent with standard R9.4.1 flow cells. Higher assembly contiguity has also been reported, including N50 values of up to 4.67 Mb and total assembly lengths of 1.30 Gb [19], compared with the values obtained in this study (N50 = 44,493 bp; total length ≈ 316 Mb). These differences likely reflect variations in sequencing depth, flow-cell chemistry, and genome size. In addition, haplotype collapsing has been identified as a major challenge in long-read assemblies of non-model and polyploid genomes [20], which may have contributed to the duplication ratio of 1.259 observed in our assembly.

To progressively raise the quality of the *Musa paradisiaca* L. genome towards Q1 reference standards, three complementary steps are proposed for subsequent releases of this resource: (i) base-level polishing with Medaka (and, when Illumina short reads are also generated, Pilon) to correct residual homopolymer and indel errors typical of Nanopore assemblies [21]; (ii) gene-space completeness assessment with BUSCO against the *embryophyta_odb10* lineage dataset, which provides an orthology-based completeness percentage comparable across *Musaceae* assemblies [22]; and (iii) Hi-C scaffolding to reconstruct chromosome-scale pseudomolecules. The integration of these steps—already standard in reference plant genome pipelines [11,12]—is expected to substantially increase both contig N50 and genome fraction in the next release of this assembly.

### 3.2. Structural Features of MpPPO1 and MpPPO2

The two PPO-coding genes identified in M. paradisiaca (MpPPO1, 1911 bp/533 aa; MpPPO2, 1753 bp/555 aa) fall within the size range commonly reported for plant PPO genes, which typically comprise coding sequences of approximately 1800 bp and precursor proteins of around 600 amino acids [23]. Both genes were intronless, a characteristic shared by most PPO family members described in plants [9,24]. Nevertheless, exceptions have been reported in several species, including banana, wheat, and olive, where PPO genes exhibit variable exon–intron structures [6,24], highlighting the evolutionary diversity of this gene family.

Intronless gene structures have been proposed to facilitate rapid mRNA production and stress-responsive gene expression [3]. Under this hypothesis, the intronless architecture of *MpPPO1* and *MpPPO2* may be compatible with rapid transcriptional responses during fruit development or tissue damage. However, direct functional implications for physiological maturity, postharvest ripening, PPO activity, or enzymatic browning require phenotypic and biochemical validation.

### 3.3. Evolutionary Relationships Within the Musaceae PPO Family

Phylogenetic reconstruction placed both *MpPPO1* and *MpPPO2* within the *M. acuminata* Colla subsp. malaccensis clade, consistent with the AAB hybrid origin of *M. paradisiaca* L. [13,14]. However, the greater phylogenetic distance of *MpPPO2* relative to *MpPPO1* suggests divergent evolutionary trajectories between the two paralogues. Similar patterns of diversification have been reported in PPO gene families from several plant species, where multiple PPO members form distinct subclades and exhibit structural and functional variation [7,8,10]. These observations support the hypothesis that plant PPO families have undergone lineage-specific duplication events followed by subfunctionalization, consistent with the proposed expansion model [4].

The identification of only two PPO genes in our *M. paradisiaca* L. assembly—compared to the ten *MaPPO* genes reported in *M. acuminata* Colla [10]—should therefore be interpreted with caution: the 42.86% genome fraction recovered suggests that additional PPO loci may reside in unassembled regions of the genome. A more comprehensive characterization of the PPO family in *M. paradisiaca* L. will require deeper sequencing, improved chemistry (e.g., R10.4.1), and the use of hybrid assembly strategies.

### 3.4. Differential Expression of MpPPO1 and MpPPO2 Across Green-Fruit Developmental Stages

The qRT-PCR analysis revealed contrasting expression profiles for the two paralogues across the field-defined green-fruit developmental stages. *MpPPO1* exhibited significantly higher expression at the physiologically mature-green stage (2^−ΔΔCT^ = 11.77; one-way ANOVA F2,6 = 14.22, *p* = 0.0053; Tukey HSD *p* = 0.022 versus early and *p* = 0.005 versus intermediate), corresponding to an approximately 23-fold increase relative to the intermediate stage and an 11.8-fold increase relative to the early-stage calibrator. In contrast, *MpPPO2* displayed only a non-significant trend toward maximal expression at the intermediate stage (2^−ΔΔCT^ = 1.759; F2,6 = 1.88, *p* = 0.232), with no significant pairwise contrast after Tukey HSD correction. The contrasting profiles, observed in the same biological samples and normalized against the same reference gene, provide evidence of transcriptional divergence between *MpPPO1* and *MpPPO2*. They do not, by themselves, establish functional divergence or subfunctionalization.

The duplication-degeneration-complementation model provides a possible theoretical framework for divergence between duplicated genes [25]. Nevertheless, the present data are limited to phylogenetic position and relative expression in three field-defined developmental categories. The higher *MpPPO1* expression at the physiologically mature-green stage and the non-significant *MpPPO2* profile could reflect differences in developmental regulation, physiological status, field conditions, or postharvest handling. Because the fruits were not followed through a controlled ripening sequence, these results cannot be used to infer ripening-specific regulatory cues or to assign distinct developmental or defence functions to either paralogue.

Temporal changes in PPO expression during fruit development, ripening, and browning have been reported in several fleshy fruits [26], although contrasting patterns, including reduced PPO expression during ripening, have also been described in banana and other species [27,28]. The higher *MpPPO1* expression observed in the field-classified physiologically mature-green fruits is compatible with regulation during late fruit development, but direct comparison with postharvest ripening studies should be made cautiously. In the absence of a controlled ripening series and direct measurements of maturity and browning, the present results cannot establish that *MpPPO1* is activated by ripening or is the primary PPO responsible for the browning phenotype.

From a biochemical perspective, PPO-mediated browning requires the interaction of PPO enzymes with phenolic substrates and oxygen after cellular compartmentalization has been disrupted. Changes in membrane integrity, phenolic-substrate availability, tissue texture, and oxidative status during development and ripening may affect quinone formation and subsequent pigment polymerization [1,3,4]. However, PPO activity, phenolic content, tissue colour, and browning intensity were not measured in this study. Consequently, the *MpPPO1* expression profile should be considered a molecular observation that requires biochemical and phenotypic validation.

Two observations—the statistically supported increase in *MpPPO1* transcript abundance at the physiologically mature-green stage and its phylogenetic proximity to characterized *M. acuminata* PPO sequences—identify *MpPPO1* as a candidate for future functional investigation. They do not identify it as the principal molecular driver of postharvest enzymatic browning. Likewise, the non-significant expression profile of *MpPPO2* is insufficient to assign a defence- or stress-specific function. Functional roles should be tested through measurements of PPO activity and browning, gene-silencing or gene-editing experiments, and evaluation under controlled developmental and postharvest conditions.

### 3.5. Biological and Biotechnological Implications: MpPPO1 as a Candidate for Functional Validation

The significantly higher *MpPPO1* expression observed in physiologically mature-green fruits makes this locus a relevant candidate for subsequent functional studies in Ecuadorian *M. paradisiaca* L. Previous research has shown that a limited number of PPO isoforms can account for a substantial proportion of PPO activity in some crop tissues, making individual paralogues potential targets for RNAi- or CRISPR/Cas9-based strategies [29]. PPO activity may also contribute to pathogen resistance [24], and non-browning potato varieties developed through PPO gene silencing illustrate the feasibility of paralogue-specific approaches [30]. Nevertheless, the current expression data do not demonstrate that *MpPPO1* controls PPO activity or browning in banana.

In this theoretical context, the single-exon structure of *MpPPO1* and its high sequence identity to characterized *M. acuminata* PPOs may facilitate the design of future functional assays, including gene-editing experiments [31,32], However, *MpPPO1* should not yet be prioritized as a crop-improvement target solely on the basis of its expression profile. Validation should first establish whether altering *MpPPO1* changes PPO activity, browning intensity, fruit quality, and disease responses. Similarly, no defence function should be assigned to *MpPPO2* without experimental evidence. Any future simultaneous or paralogue-specific editing strategy should therefore include off-target assessment and evaluation of both postharvest quality and plant defence.

### 3.6. Limitations of the Study

Several limitations of the present work should be acknowledged. First, the genome assembly covers 42.86% of the *M. acuminata* Colla DH-Pahang reference and was generated at approximately 5× coverage on a single FLO-MIN106 R9.4.1 flow cell; consequently, BUSCO benchmarking and Medaka polishing were not performed, and additional PPO paralogues may reside in unassembled or low-quality regions. The two loci identified here should therefore be treated as a lower bound for the *M. paradisiaca* L. PPO repertoire. Second, the analysis was performed on a single genotype from one Ecuadorian farm, and expression patterns may differ among cultivars, cultivation conditions, or harvest years. Third, the fruit categories were assigned using a qualitative visual field index based on finger filling, fruit size and shape, and angularity, without quantitative measurements of days from flowering or bunch emergence, firmness, soluble solids, instrumental colour, respiration, or ethylene production. Therefore, the categories represent field-defined green-fruit developmental stages rather than instrumentally validated physiological maturity or consumer ripeness classes. Fourth, peel tissue was frozen within 1 to 3 days after harvest under non-controlled ambient handling, which may have introduced postharvest variation in gene expression. Finally, the qRT-PCR analysis was based on three biological replicates per stage. Although the *MpPPO1* comparison was statistically significant, the sample size limited the power to resolve the more subtle *MpPPO2* pattern. These limitations restrict causal and physiological interpretation of the expression profiles.

Future studies should combine (i) deeper genome sequencing, consensus polishing, BUSCO assessment, and chromosome-scale scaffolding; (ii) fruit sampling based on a standardized harvest index that records days from flowering or bunch emergence, finger filling, angularity, dimensions, and position within the bunch; (iii) controlled postharvest ripening with standardized temperature, relative humidity, and ethylene exposure; (iv) immediate and uniform tissue freezing; (v) quantitative measurements of peel colour, firmness, soluble solids, respiration, ethylene, PPO activity, phenolic substrates, and browning intensity; (vi) larger numbers of independent bunches across harvest seasons; and (vii) functional validation through gene silencing or CRISPR/Cas9-based editing. These steps are required to determine whether *MpPPO1* has a direct causal role in PPO activity or enzymatic browning.

## 4. Materials and Methods

### 4.1. Plant Material, Study Site, and Classification of Fruit Developmental Stages

Leaf tissue of *M. paradisiaca* L. was collected from a commercial plantation affiliated with the Universidad Técnica Estatal de Quevedo (UTEQ), located in Quevedo, Los Ríos Province, Ecuador. Samples were flash-stored and transported under cold-chain conditions to the Molecular Biology Laboratory of UTEQ and to the Faculty of Experimental Sciences of the Universidad de Jaén (Campus Las Lagunillas s/n, 23071 Jaén, Spain), where they were maintained at −80 °C until processing. For gene-expression analysis, fruit samples were collected from the same orchard at three field-defined green-fruit developmental stages: early, intermediate, and physiologically mature-green, with three biological replicates per stage. All fruits were harvested directly from the plant. Classification was performed in the field by an experienced local grower using a qualitative visual harvest index based on the degree of finger filling, characteristic fruit size and shape, reduction in external angularity, and complementary visual indicators of bunch development. Early-stage fruits showed less finger filling and pronounced angularity; intermediate-stage fruits showed partial filling and greater development while retaining visible angularity; and physiologically mature-green fruits showed full finger filling, characteristic commercial size and shape, and clearly reduced angularity. Approximate time after sucker planting and the onset of leaf drying were retained only as contextual field observations and were not treated as independent indices of physiological fruit maturity.

Fruit collection took place on 16 April 2024 in a single sampling campaign. Because the plantation follows a continuous, staggered planting cycle, bunches corresponding to the three green-fruit developmental stages were simultaneously present in the field. Each hand was detached manually from the bunch using a machete, following standard local commercial practice. After cutting, hands were transported under ambient, non-refrigerated conditions to the Molecular Biology Laboratory of UTEQ, where peel tissue was excised and frozen at −80 °C within 1 to 3 days of harvest. Temperature and relative humidity during this interval were not recorded, and no controlled ripening chamber, ethylene treatment, or extended storage period was applied. Accordingly, the three categories describe fruit development at field harvest and do not represent sequential stages of controlled postharvest ripening. In this manuscript, “physiologically mature-green” refers to fruit classified in the field as having completed sufficient development for commercial harvest, whereas “ripe” is reserved for consumption maturity after postharvest ripening and is not used to describe the experimental samples. Representative photographs are shown in Figure 2 for descriptive purposes. Fruit mass, instrumental peel colour, firmness, total soluble solids, pulp-to-peel ratio, ethylene production, and respiration rate were not measured. Therefore, classification relied on the qualitative field index described above [33,34], and the absence of quantitative physicochemical and physiological maturity measurements is recognized as a limitation.

### 4.2. Genomic DNA Extraction and Oxford Nanopore Sequencing

High-molecular-weight (HMW) genomic DNA was extracted from frozen leaf tissue using the Illustra Nucleon Phytopure Genomic DNA Extraction Kit (Cytiva, Amersham Place, Little Chalfont, Buckinghamshire, United Kingdom), following the manufacturer’s instructions. The recovery of HMW DNA is a prerequisite for effective long-read sequencing of plant genomes [12]. DNA concentration was quantified with a Qubit 4 fluorometer using the dsDNA Broad Range assay (Thermo Fisher Scientific, Waltham, MA, USA); purity was assessed by microvolume spectrophotometry (NanoDrop; A_260_/A_280_ and A_260_/A_230_ ratios); and DNA integrity and fragment-size distribution were verified by 0.8% (*w*/*v*) agarose gel electrophoresis under low-voltage conditions to preserve HMW fragments.

Long-read sequencing of the *M. paradisiaca* L. genome was performed on the MinION Mk1B platform (device identifier MN28726; Oxford Nanopore Technologies, Oxford, UK) using an R9.4.1 flow cell (FLO-MIN106) and the Rapid Sequencing Kit SQK-RAD004, according to the manufacturer’s protocol. The SQK-RAD004 chemistry employs a transposase-mediated tagmentation step that ligates sequencing adapters in a single 10 min reaction; this rapid workflow trades off some output yield for reduced library-preparation time and a lower starting-material requirement and is well established for plant genomes when HMW DNA is available [16]. The MinION platform identifies nucleotides through real-time changes in electrical conductivity as single-stranded DNA translocates through a biological nanopore [15]. After obtaining the report from MinkNOW with a total of 1.27 × 10^6^ reads yielding approximately 4.79 Gb of raw signal (3.7 Gb after base identification), the raw signal data in FAST5 format were converted to FASTQ nucleotide sequences from base identification using Guppy v6.4.6 in high-precision mode (HAC model dna_r9.4.1_450bps_hac.cfg). Reads shorter than 1000 bp or with a median quality score below Q8 were discarded using NanoFilt (https://github.com/wdecoster/nanofilt, accessed on 21 July 2026) before further analysis.

### 4.3. Genome Assembly and Quality Assessment

Filtered reads were uploaded to the Galaxy Europe server (https://usegalaxy.eu). Read-quality metrics (per-read quality score, length distribution, cumulative yield, N50 of the read set) were computed with NanoPlot v1.41. De novo assembly was performed with Flye (Galaxy version 2.9.6+galaxy0) using the nano raw input mode, an expected genome size of 550 Mb—set by reference to the size of the *M. acuminata* Colla DH-Pahang assembly [12]—with default values for the remaining parameters and a single internal polishing iteration enabled [17]. Theoretical sequencing coverage was estimated at ~5× based on the total yield of filtered reads and the expected genome size; this depth, although below the ≥30× generally recommended for chromosome-scale assemblies, is sufficient for unambiguous gene-level identification when the target loci are intronless and present in low copy numbers, as is the case for *MpPPO1* and *MpPPO2* (Section 2.3).

Assembly quality was evaluated with QUAST v5.2 using the chromosome-scale *M. acuminata* DH-Pahang reference genome retrieved from the Banana Genome Hub (https://banana-genome-hub.southgreen.fr). Both reference-free metrics (number of contigs, largest contig, N50, N90, auN, L50, L90, GC content) and reference-based metrics (genome fraction, duplication ratio, total aligned length, largest alignment) were computed and are summarized in Table 1. The use of a chromosome-scale reference additionally supports the downstream analysis of inter-individual variability and evolutionary divergence [35].

BUSCO-based gene-space completeness assessment (*embryophyta_odb10* lineage dataset) and Medaka post-assembly base-level polishing were not executed in the present study because the ~5× coverage achieved on a single MinION flow cell falls below the depth at which both procedures yield robust and biologically interpretable estimates. BUSCO scores derived from low-coverage assemblies are systematically deflated by partial gene reconstruction rather than by true gene-space loss, and Medaka polishing requires a minimum effective per-base coverage of ~20× to converge on genuine consensus corrections, below which it can introduce, rather than remove, indel artefacts [21]. Importantly, the unambiguous identification of the two PPO loci reported here does not depend on these completeness metrics: both *MpPPO1* and *MpPPO2* are intronless single-copy genes whose full coding sequence was recovered, without internal gaps, on a single contig per locus, with >95% nucleotide identity to the orthologous reference sequences from the *M. acuminata* Colla subsp. *malaccensis* clade (Section 2.3 and Section 2.4). Deeper sequencing using R10.4.1 chemistry, followed by Medaka polishing and BUSCO benchmarking, is planned for the next release of this assembly (Section 3.6).

### 4.4. Identification and Annotation of PPO-Coding Genes

PPO-coding genes were identified by local BLASTn alignment (implemented in Galaxy Europe) between the *M. paradisiaca* L. assembly and the *M. acuminata* Colla PPO reference sequences reported in [10]. Contigs containing significant hits (E-value < 1 × 10^−50^, query coverage ≥ 80%) were retrieved and the gene structure (exons and introns) was predicted with Augustus v3.3.3 (https://bioinf.uni-greifswald.de/augustus, accessed on 29 September 2024)). Predicted coding sequences (CDS) and translated protein sequences were curated and deposited in GenBank under accession numbers PX565008 (*MpPPO1*) and PX565009 (*MpPPO2*). The complete assembly was deposited under accession JBUBWB000000000.1.

### 4.5. Phylogenetic Analysis

A set of 31 PPO nucleotide sequences from agronomically relevant species—including wheat, barley, rice, banana, apple, strawberry, tomato, eggplant, and potato—was retrieved from the NCBI nucleotide database. Multiple sequence alignment was performed using the MUSCLE algorithm implemented in MEGA v12, with default parameters. A phylogenetic tree was reconstructed using the Neighbor-Joining (NJ) method with 500 bootstrap replicates. *MpPPO1* and *MpPPO2* were included in the alignment together with all recovered homologues.

### 4.6. RNA Extraction and cDNA Synthesis

Total RNA was extracted from the peel of fruits at the early, intermediate, and physiologically mature-green developmental stages (three biological replicates per stage) using the E.Z.N.A.^®^ Plant RNA Kit (Omega Bio-tek, Norcross, GA, USA), which includes tissue homogenization, cell lysis, and column-based RNA purification. An on-column DNase I treatment was performed during extraction to eliminate residual genomic DNA. RNA concentration was measured by microvolume spectrophotometry (NanoDrop); purity was verified by the A260/A280 and A260/A230 ratios, and integrity was assessed on 1% (*w*/*v*) denaturing agarose gels using the integrity of the 28S and 18S ribosomal bands.

Reverse transcription was performed from 1 µg of total RNA per reaction using the OneScript^®^ Plus cDNA Synthesis Kit (Applied Biological Materials, Richmond, BC, Canada) and the SuperScript™ III Reverse Transcriptase Kit (Invitrogen, Carlsbad, CA, USA) with random hexamer primers, according to the manufacturers’ recommendations. cDNA integrity and concentration were verified by microvolume spectrophotometry and agarose gel electrophoresis. The resulting cDNA was diluted to ~100 ng µL^−1^ for endpoint PCR validation of primer amplification, and subsequently to 10 ng µL^−1^ for qPCR quantification.

### 4.7. Primer Design and Endpoint-PCR Validation

Primer pairs for MpPPO1 and MpPPO2 were adapted from published Musa PPO genes [10] and validated in silico using NCBI Primer-BLAST against the assembled sequences. Primer sequences are listed in Table 3.

Endpoint PCR was performed to confirm amplification of the expected products (112 bp for *MpPPO1* and 237 bp for *MpPPO2*) prior to qPCR quantification. In parallel, a chloroplast *ndhB* fragment (~721 bp) was amplified by endpoint PCR from each cDNA preparation as an internal quality control of reverse-transcription efficiency and to confirm the absence of PCR inhibitors; this control is shown in Figure 4A,B.

### 4.8. Quantitative Real-Time PCR (qRT-PCR) and MIQE Compliance

Quantitative PCR was carried out using PowerUp™ SYBR™ Green Master Mix (Applied Biosystems, Foster City, CA, USA) in a final reaction volume of 10 µL, following the manufacturer’s protocol, under the thermocycling conditions detailed in Table 4. Each biological replicate was analyzed in technical triplicate, and ROX (carboxy-X-rhodamine) was used as the passive reference dye, as visible in the amplification traces of all qPCR runs (Supplementary Materials).

The gene encoding Ribosomal Protein S2 (*RPS2*) was selected as the endogenous reference gene. *RPS2* encodes the universally conserved 40S ribosomal protein uS5 [36,37] and consequently fulfills the canonical criteria of a constitutive housekeeping gene: it participates in a fundamental cellular process (mRNA translation), is expressed in every cell type, and exhibits stable transcript abundance across developmental stages, tissues, and stress conditions. In *Musa* spp., the suitability of *RPS2* as a stable internal control for normalization of qRT-PCR data has been documented in systematic reference-gene validation studies [36,38], in which ribosomal protein–encoding transcripts consistently rank among the most stably expressed loci across tissues, developmental stages, ripening stages and biotic-stress treatments. This evidence justifies its use as the reference gene for the present analysis of *MpPPO1* and *MpPPO2* expression during banana fruit ripening.

According to MIQE standards [39], the amplification efficiency of the primers (E) was determined using five-point standard curves prepared by serial 1:10 dilutions of a combined cDNA template (10 to 10^−4^ ng µL^−1^). The slope of the linear regression between Ct and the log10 cDNA concentration was used to calculate E using the formula E = 10(−1/slope) − 1. The criteria for accepting the result were R^2^ ≥ 0.98 and E = 90 to 110%.

Amplicon specificity was confirmed at the end of every qPCR run by melting-curve analysis (65 → 95 °C, 0.5 °C increments of 5 s). For each primer pair—*MpPPO1*, *MpPPO2* and the *RPS2* reference gene—the dissociation profile displayed a single, sharp peak at the expected melting temperature in every biological replicate (Supplementary Materials), with no secondary peaks at lower temperatures. This pattern confirms the absence of primer-dimer artifacts and non-specific amplification products and demonstrates that each Ct value reported in the present study originates from a single, target-specific amplicon, in full compliance with the MIQE specificity criteria. All assays were performed using template-free controls (TFCs) and reverse transcriptase-free controls (−RTs), which consistently generated Ct values above the 40-cycle threshold (Table 4). This eliminated the possibility of contamination in the master mixes and residual carryover of genomic DNA. Ct values exceeding 40 cycles were excluded from the analysis because they were considered non-amplified.

The early green-fruit developmental stage was used as the calibrator for the target genes, and *RPS2* was used as the reference gene to calculate relative expression using the 2^−ΔΔCT^ method [40].

### 4.9. Statistical Analysis

Relative expression data were presented as the mean ± standard deviation (SD) from three biological replicates per developmental stage. Statistical comparisons were performed on ΔCt values rather than on the non-normally distributed 2^−ΔΔCT^ values, in accordance with established recommendations for qPCR data analysis [39,41]. Differences among the early, intermediate, and physiologically mature-green stages were evaluated using one-way analysis of variance (ANOVA), followed by Tukey’s Honestly Significant Difference (HSD) post hoc test for pairwise comparisons. Normality and homoscedasticity of residuals were evaluated using the Shapiro–Wilk and Levene tests, respectively. A significance threshold of *p* < 0.05 was adopted. All statistical analyses were carried out in Python v3.11 using the scipy.stats module v1.11, and graphs were generated in R v4.3 using ggplot2. Ct values above 40 cycles were considered non-amplification and excluded.

## 5. Conclusions

This study reports a preliminary de novo genome assembly of *M. paradisiaca* L. from Ecuadorian germplasm generated by Oxford Nanopore long-read sequencing and Flye assembly. The assembly comprised 13,239 contigs with a total length of 315,948,728 bp, an N50 of 44,493 bp, and a genome fraction of 42.86% relative to the *M. acuminata* Colla DH-Pahang reference. Two intronless PPO-coding genes, *MpPPO1* (1911 bp; 533 aa) and *MpPPO2* (1753 bp; 555 aa), were identified, annotated, and deposited in GenBank under accessions PX565008 and PX565009. qRT-PCR revealed significantly higher *MpPPO1* expression at the field-classified physiologically mature-green stage, whereas *MpPPO2* showed a non-significant maximum at the intermediate stage. These results support transcriptional divergence between the two loci and identify *MpPPO1* as a candidate for future functional investigation. However, because the fruit stages were defined by a qualitative field harvest index and neither controlled postharvest ripening nor quantitative maturity, PPO activity, or browning measurements were performed, the data do not establish ripening-induced activation, functional subfunctionalization, or a causal role for *MpPPO1* in postharvest browning. Functional and biochemical validation under standardized developmental and postharvest conditions is required before proposing *MpPPO1* as a gene-editing target.

## Figures and Tables

**Table 1:** Assembly and quality control metrics of the *M. paradisiaca* L. genome obtained with Flye and evaluated with QUAST against the *M. acuminata* Colla DH-Pahang reference genome.

Metric	Value
**Reference-based statistics**	
Genome fraction (%)	42.864
Duplication ratio	1.259
Largest alignment (bp)	157,249
Total aligned length (bp)	179,788,021
**Reference-free statistics**	
Number of contigs (≥0 bp)	13,239
Number of contigs (≥1000 bp)	12,887
Largest contig (bp)	411,031
Total length (bp)	315,948,728
Total length, ≥1000 bp	315,721,432
N50 (bp)	44,493
N90 (bp)	11,434
auN	62,379
L50	1946
L90	7458
GC content (%)	39.56

Note. BUSCO completeness scores and Medaka post-assembly polishing metrics are not reported here because the present dataset was generated as an initial low-coverage draft (~5×) on a single MinION Mk1B flow cell. Because low coverage may underestimate gene-space completeness, the absence of BUSCO and Medaka results is acknowledged as a methodological limitation. The identification of MpPPO1 and MpPPO2 should therefore be interpreted at the gene level within the recovered assembly, and not as evidence of complete recovery of the PPO family or the whole genome.

**Table 2:** Structural characterization of the *MpPPO1* and *MpPPO2* genes identified in *M. paradisiaca* L.

Gene	Version	Protein (aa)	CDS (bp)	Gene (bp)	Introns	Accession
*MpPPO1*	1	533	1911	1911	0	PX565008
*MpPPO2*	1	555	1753	1753	0	PX565009

Note. CDS: Coding DNA Sequences.

**Table 3:** Primer sequences used for the expression analysis of MpPPO1 and MpPPO2 genes.

Gene	Forward Primer (5′→3′)	Reverse Primer (5′→3′)	Amplicon (bp)
*MpPPO1*	CTTCACTCAGCATGCCAACG	CCATGGCAGGAAGAGCCATGA	112
*MpPPO2*	CTTCTGCCACCATTGCAATGTC	GAGGCAGTCGCTCATCTTG	237
*RPS2*	TAGGGATTCCGACGATTTGTTT	TAGCGTCATCATTGGCTGGGA	84

*RPS2* primers adapted from [36].

**Table 4:** Thermocycling conditions for qPCR amplification of MpPPO1 and MpPPO2.

Step	Temperature (°C)	Time	Cycles
UDG activation	50	2 min	1
Dual-Lock™ DNA polymerase activation	95	2 min	1
Denaturation	95	15 s	40
Annealing/Extension	58	1 min	40

## Data Availability

The datasets generated and analyzed in this study are publicly available in the National Center for Biotechnology Information (NCBI). The complete genome assembly of *Musa paradisiaca* has been deposited in DDBJ/ENA/GenBank under accession number JBUBWB000000000.1 https://www.ncbi.nlm.nih.gov/nuccore/JBUBWB000000000.1 (accessed on 18 December 2025). The annotated sequences of the *MpPPO1* and *MpPPO2* genes have been deposited under accession numbers PX565008 https://www.ncbi.nlm.nih.gov/nuccore/PX565008 and PX565009 https://www.ncbi.nlm.nih.gov/nuccore/PX565009 accessed on 12 November 2025, respectively.

## Supplementary Materials

The following supporting information can be downloaded at: https://www.mdpi.com/article/10.3390/plants15152329/s1, Supplementary material; Table S1: Relative expression of the MpPPO1 gene (2^−ΔΔCT^ method) across three phenological stages of *M. paradisiaca* L. fruit, by biological replicate. Table S2. Relative expression of the MpPPO2 gene (2^−ΔΔCT^ method) across three phenological stages of *M. paradisiaca* L. fruit, by biological replicate.

## References

[1] Morante-Carriel J., Obrebska A., Rodríguez J., Carranza Patiño M., Pico-Saltos R., Bru R. (2014). Distribución, localización e inhibidores de las polifenol oxidasas en frutos y vegetales usados como alimento. Cienc. Tecnol..

[2] Nascimento M.B., Alencar J.C.G., Paulino B.N., Nascimento J.C.N., Ferreira T.R., Batista A.S., Nascimento M.M., Soares S.E., Mesquita P.R.R. (2025). Functional and technological potential of by-products from the cocoa (*Theobroma cacao* L.) production chain. Food Chem..

[3] He F., Shi Y.J., Zhao Q., Zhao K.J., Cui X.L., Chen L.H., Yang H.B., Zhang F., Mi J.X., Huang J.L. (2021). Genome-wide investigation and expression profiling of polyphenol oxidase (PPO) family genes uncover likely functions in organ development and stress responses in *Populus trichocarpa*. BMC Genom..

[4] Tran L.T., Taylor J.S., Constabel C.P. (2012). The polyphenol oxidase gene family in land plants: Lineage-specific duplication and expansion. BMC Genom..

[5] Wang C., Meng L., Zhang G., Yang X., Pang B., Cheng J., He B., Sun F. (2024). Unraveling crop enzymatic browning through integrated omics. Front. Plant Sci..

[6] Liu Q., Wang C., Cui Q., Fan Y., Zhang J., Rao G. (2023). Genome-Wide Analysis of the Polyphenol Oxidase Gene Family in *Olea europaea* Provides Insights into the Mechanism of Enzymatic Browning in Olive Fruit. Antioxidants.

[7] Zhao Z., Wang F., Deng M., Fan G. (2024). Identification and Analysis of PPO Gene Family Members in *Paulownia fortunei*. Plants.

[8] Pompili V., Mazzocchi E., Moglia A., Acquadro A., Comino C., Rotino G.L., Lanteri S. (2023). Structural and expression analysis of polyphenol oxidases potentially involved in globe artichoke (*C. cardunculus* var. *scolymus* L.) Tissue browning. Sci. Rep..

[9] Guo M., Tang Y., Yang Y., Luo J., Gao J. (2025). Identification and Expression Analysis of Polyphenol Oxidase Gene Family Members in Response to Wound Stress in Lettuce (*Lactuca sativa* L.). Plants.

[10] Qin F., Hu C., Dou T., Sheng O., Yang Q., Deng G., He W., Gao H., Li C., Dong T. (2023). Genome-wide analysis of the polyphenol oxidase gene family reveals that MaPPO1 and MaPPO6 are the main contributors to fruit browning in *Musa acuminate*. Front. Plant Sci..

[11] Wang Z.-F., Rouard M., Droc G., Heslop-Harrison P., Ge X.-J. (2023). Genome assembly of *Musa beccarii* shows extensive chromosomal rearrangements and genome expansion during evolution of Musaceae genomes. GigaScience.

[12] Belser C., Baurens F.-C., Noel B., Martin G., Cruaud C., Istace B., Yahiaoui N., Labadie K., Hřibová E., Doležel J. (2021). Telomere-to-telomere gapless chromosomes of banana using nanopore sequencing. Commun. Biol..

[13] Cenci A., Sardos J., Hueber Y., Martin G., Breton C., Roux N., Swennen R., Carpentier S.C., Rouard M. (2021). Unravelling the complex story of intergenomic recombination in ABB allotriploid bananas. Ann. Bot..

[14] Borah S., Bora D., Bhorali P. (2022). Infection by *Pseudocercospora musae* leads to an early reprogramming of the *Musa paradisiaca* defense transcriptome. 3 Biotech.

[15] Lu H., Giordano F., Ning Z. (2016). Oxford Nanopore MinION Sequencing and Genome Assembly. Genom. Proteom. Bioinform..

[16] Murigneux V., Rai S.K., Furtado A., Bruxner T.J.C., Tian W., Harliwong I., Wei H., Yang B., Ye Q., Anderson E. (2020). Comparison of long-read methods for sequencing and assembly of a plant genome. GigaScience.

[17] Kolmogorov M., Yuan J., Lin Y., Pevzner P.A. (2019). Assembly of long, error-prone reads using repeat graphs. Nat. Biotechnol..

[18] Choi J.Y., Lye Z.N., Groen S.C., Dai X., Rughani P., Zaaijer S., Harrington E.D., Juul S., Purugganan M.D. (2020). Nanopore sequencing-based genome assembly and evolutionary genomics of circum-basmati rice. Genome Biol..

[19] Rao G., Zhang J., Liu X., Lin C., Xin H., Xue L., Wang C. (2021). De novo assembly of a new *Olea europaea* genome accession using nanopore sequencing. Hortic. Res..

[20] Guiglielmoni N., Houtain A., Derzelle A., Van Doninck K., Flot J.-F. (2021). Overcoming uncollapsed haplotypes in long-read assemblies of non-model organisms. BMC Bioinform..

[21] Wick R.R., Holt K.E. (2022). Polypolish: Short-read polishing of long-read bacterial genome assemblies. PLoS Comput. Biol..

[22] Manni M., Berkeley M.R., Seppey M., Simão F.A., Zdobnov E.M. (2021). BUSCO Update: Novel and Streamlined Workflows along with Broader and Deeper Phylogenetic Coverage for Scoring of Eukaryotic, Prokaryotic, and Viral Genomes. Mol. Biol. Evol..

[23] Mishra B.B. (2016). Polyphonel Oxidases: Biochemical and Molecular Characterization, Distribution, Role and its Control. Enzym. Eng..

[24] Newman S.M., Eannetta N.T., Yu H., Prince J.P., Carmen de Vicente M., Tanksley S.D., Steffens J.C. (1993). Organisation of the tomato polyphenol oxidase gene family. Plant Mol. Biol..

[25] Force A., Lynch M., Pickett F.B., Amores A., Yan Y.-l., Postlethwait J. (1999). Preservation of Duplicate Genes by Complementary, Degenerative Mutations. Genetics.

[26] Wang J., Liu B., Xiao Q., Li H., Sun J. (2014). Cloning and expression analysis of litchi (*Litchi Chinensis* Sonn.) polyphenol oxidase gene and relationship with postharvest pericarp browning. PLoS ONE.

[27] Gooding P.S., Bird C., Robinson S.P. (2001). Molecular cloning and characterisation of banana fruit polyphenol oxidase. Planta.

[28] Chevalier T., de Rigal D., Mbéguié A.M.D., Gauillard F., Richard-Forget F., Fils-Lycaon B.R. (1999). Molecular cloning and characterization of apricot fruit polyphenol oxidase. Plant Physiol..

[29] Bøjer Rasmussen C., Enghild J.J., Scavenius C. (2021). Identification of polyphenol oxidases in potato tuber (*Solanum tuberosum*) and purification and characterization of the major polyphenol oxidases. Food Chem..

[30] Halterman D., Guenthner J., Collinge S., Butler N., Douches D. (2016). Biotech Potatoes in the 21st Century: 20 Years Since the First Biotech Potato. Am. J. Potato Res..

[31] Kaur N., Alok A., Shivani, Kumar P., Kaur N., Awasthi P., Chaturvedi S., Pandey P., Pandey A., Pandey A.K. (2020). CRISPR/Cas9 directed editing of lycopene epsilon-cyclase modulates metabolic flux for β-carotene biosynthesis in banana fruit. Metab. Eng..

[32] Tripathi J.N., Ntui V.O., Ron M., Muiruri S.K., Britt A., Tripathi L. (2019). CRISPR/Cas9 editing of endogenous banana streak virus in the B genome of *Musa* spp. overcomes a major challenge in banana breeding. Commun. Biol..

[33] Kolombia Y.A., Swennen R. (2025). Standard Operating Procedure (SOP) for Banana and Plantain Harvesting (SOP17).

[34] Robinson J.C., Galán Saúco V. (2010). Bananas and Plantains.

[35] Motazedi E., Finkers R., Maliepaard C., de Ridder D. (2018). Exploiting next-generation sequencing to solve the haplotyping puzzle in polyploids: A simulation study. Brief. Bioinform..

[36] Chen L., Zhong H.-y., Kuang J.-F., Li J.-G., Lu W.-J., Chen J.-Y. (2011). Validation of reference genes for RT-qPCR studies of gene expression in banana fruit under different experimental conditions. Planta.

[37] Dionne K.L., Bergeron D., Landry-Voyer A.-M., Bachand F. (2019). The 40S ribosomal protein uS5 (RPS2) assembles into an extraribosomal complex with human ZNF277 that competes with the PRMT3–uS5 interaction. J. Biol. Chem..

[38] Podevin N., Krauss A., Henry I., Swennen R., Remy S. (2012). Selection and validation of reference genes for quantitative RT-PCR expression studies of the non-model crop *Musa*. Mol. Breed..

[39] Bustin S.A., Benes V., Garson J.A., Hellemans J., Huggett J., Kubista M., Mueller R., Nolan T., Pfaffl M.W., Shipley G.L. (2009). The MIQE Guidelines: Minimum Information for Publication of Quantitative Real-Time PCR Experiments. Clin. Chem..

[40] Livak K.J., Schmittgen T.D. (2001). Analysis of Relative Gene Expression Data Using Real-Time Quantitative PCR and the 2^−ΔΔCT^ Method. Methods.

[41] Schmittgen T.D., Livak K.J. (2008). Analyzing real-time PCR data by the comparative CT method. Nat. Protoc..

