# Supplementary material for: Draft Genome Assembly and Differential Expression of MpPPO1 and MpPPO2 Across Green-Fruit Development in Ecuadorian Musa paradisiaca L
Source: Plants (Basel). 2026 Jul 29;15(15):2329. doi: 10.3390/plants15152329 (PMC13467689; doi:10.3390/plants15152329)
Supplement: Supplementary file 1 [file plants-15-02329-s001.zip › plants-4374964-supplementary.pdf]

# Supplementary Materials

### Table S1. Relative expression of the MpPPO1 gene ($2^{-\Delta\Delta CT}$ method) across three phenological stages of M*. paradisiaca* L. fruit, by biological replicate.

| Stage | Rep | Ct RPS2 | Mean Ct RPS2 | Ct target | Mean Ct target | ΔCt | ΔΔCt | 2⁻ΔΔCt | Mean | SD |
|---|---|---|---|---|---|---|---|---|---|---|
| Early | 1 | 17.551 | 17.874 | 32.199 | 32.447 | 14.573 | 0.000 | 1.000 | | |
| Early | 2 | 17.012 | | 31.973 | | | | | | |
| Early | 3 | 19.059 | | 33.168 | | | | | | |
| Intermediate Stages | 1 | 21.129 | 19.025 | 37.691 | 34.574 | 15.549 | 0.976 | 0.508 | | |
| Intermediate Stages | 2 | 18.473 | | 33.129 | | | | | | |
| Intermediate Stages | 3 | 17.473 | | 32.902 | | | | | | |
| Mature-green | 1 | 20.434 | 20.270 | 31.785 | 32.262 | 11.992 | −3.557 | 11.770 | 4.426 | 6.365 |
| Mature-green | 2 | 20.574 | | 32.035 | | | | | | |
| Mature-green | 3 | 19.801 | | 32.965 | | | | | | |

*Note. Ct: cycle threshold; ΔCt: delta cycle threshold; ΔΔCt: delta-delta cycle threshold; $2^{-}$ΔΔCt: normalized relative gene expression; Ct RPS2: Ct of the reference gene RPS2. Corresponds to Figure 2B of the main text.*

### Table S2. Relative expression of the MpPPO2 gene ($2^{-\Delta\Delta CT}$ method) across three phenological stages of *M. paradisiaca* L. fruit, by biological replicate.

| Stage | Rep | Ct RPS2 | Mean Ct RPS2 | Ct target | Mean Ct target | ΔCt | ΔΔCt | 2⁻ΔΔCt | Mean | SD |
|---|---|---|---|---|---|---|---|---|---|---|
| Early | 1 | 22.215 | 20.772 | 22.113 | 21.780 | 1.008 | 0.000 | 1.000 | | |
| Early | 2 | 19.426 | | 20.887 | | | | | | |
| Early | 3 | 20.676 | | 22.340 | | | | | | |
| Intermediate Stages | 1 | 21.699 | 21.173 | 21.371 | 21.366 | 0.193 | −0.815 | 1.759 | | |
| Intermediate Stages | 2 | 20.965 | | 20.949 | | | | | | |
| Intermediate Stages | 3 | 20.855 | | 21.777 | | | | | | |
| Mature-green | 1 | 22.559 | 22.171 | 24.332 | 23.715 | 1.544 | 0.536 | 0.690 | 1.150 | 0.55 |
| Mature-green | 2 | 23.379 | | 23.902 | | | | | | |
| Mature-green | 3 | 20.574 | | 22.910 | | | | | | |

*Note. Ct: cycle threshold; ΔCt: delta cycle threshold; ΔΔCt: delta-delta cycle threshold; $2^{-}$ΔΔCt: normalized relative gene expression; Ct RPS2: Ct of the reference gene RPS2. Corresponds to Figure 4 of the main text.*
